# The Effectiveness of Adductor Canal Block Compared to Femoral Nerve Block on Readiness for Discharge in Patients Undergoing Outpatient Anterior Cruciate Ligament Reconstruction: A Multi-Center Randomized Clinical Trial

**DOI:** 10.3390/jcm12186019

**Published:** 2023-09-17

**Authors:** Werner ten Hoope, Manouk Admiraal, Jeroen Hermanides, Henning Hermanns, Markus W. Hollmann, Philipp Lirk, Gino M. M. W. Kerkhoffs, Jeroen Steens, Rienk van Beek

**Affiliations:** 1 Department of Anesthesiology, Amsterdam University Medical Center, University of Amsterdam, Meibergdreef 9, 1105 AZ Amsterdam, The Netherlands; 2 Department of Anesthesiology, Perioperative and Pain Medicine, Brigham and Women’s Hospital, Harvard Medical School, 75 Francis Street, Boston, MA 02115, USA; 3 Department of Orthopedic Surgery and Sports Medicine, Amsterdam Movement Sciences, Amsterdam University Medical Centers, Meibergdreef 9, 1105 AZ Amsterdam, The Netherlands; 4 Department of Orthopedics, Dijklander Ziekenhuis, Maelsonstraat 3, 1624 NP Hoorn, The Netherlands; 5 Department of Anesthesiology, Dijklander Ziekenhuis, Maelsonstraat 3, 1624 NP Hoorn, The Netherlands

**Keywords:** nerve block, pain, postoperative, analgesics, opioid, anterior cruciate ligament reconstruction

## Abstract

This study evaluated the effect of adductor canal block (ACB) versus femoral nerve block (FNB) on readiness for discharge in patients undergoing outpatient anterior cruciate ligament (ACL) reconstruction. We hypothesized that ACB would provide sufficient pain relief while maintaining motor strength and safety, thus allowing for earlier discharge. This was a randomized, multi-center, superiority trial. From March 2014 to July 2017, patients undergoing ACL reconstruction were enrolled. The primary outcome was the difference in readiness for discharge, defined as Post-Anesthetic Discharge Scoring System score ≥ 9. Twenty-six patients were allocated to FNB and twenty-seven to ACB. No difference in readiness for discharge was found (FNB median 1.8 (95% CI 1.0 to 3.5) vs. ACB 2.9 (1.5 to 4.7) hours, *p* = 0.3). Motor blocks and (near) falls were more frequently reported in patients with FNB vs. ACB (20 (76.9%) vs. 1 (3.7%), *p* < 0.001, and 7 (29.2%) vs. 1 (4.0%), *p* = 0.023. However, less opioids were consumed in the post-anesthesia care unit for FNB (median 3 [0, 21] vs. 15 [12, 42.5] oral morphine milligram equivalents, *p* = 0.004) for ACB. Between patients with FNB or ACB, no difference concerning readiness for discharge was found. Despite a slight reduction in opioid consumption immediately after surgery, FNB demonstrates a less favorable safety profile compared to ACB, with more motor blocks and (near) falls.

## 1. Introduction

The number of patients undergoing anterior cruciate ligament (ACL) reconstruction has risen over the past decade [1]. This procedure is typically performed in an outpatient setting, due to satisfactory functional and cost-effective outcomes [2]. For outpatient surgery, adequate pain management is crucial for timely discharge of patients. Regional anesthesia, particularly peripheral nerve blocks, has become increasingly important in optimizing postoperative pain management and expediting discharge. 

Nonetheless, guidance in this matter continues to exhibit variability. The Society for Ambulatory Anesthesia advocates for the utilization of local installation analgesia as the primary approach, suggesting the adoption of adductor canal block or femoral nerve block only when local installation is not a feasible option [3]. In contrast, a recent published network analysis, suggests the consideration of a single injection femoral nerve block combined with a sciatic nerve block, or local infiltration analgesia [4].

While femoral nerve blocks (FNB) have been associated with less pain and reduced analgesic consumption [5], there is an increased risk of motor weakness of the quadriceps muscles, and accidental falls as compared to a placebo [6]. Besides short-term effects, a decreased quadriceps strength has also been observed six weeks after ACL reconstruction in patients who had received FNB [7]. Adductor canal block (ACB) has been proposed as a safe alternative preserving motor function [8]. Several studies have reported similar pain relief and a reduction in the loss of quadriceps motor strength in favor of ACB compared to FNB [8,9,10,11,12]. However, other studies have reported more pain after ACB [13,14], or similar quadriceps muscle function between both groups [15,16,17]. 

Most of these studies base their conclusions regarding quadriceps motor strength on very specific outcome measurements, such as surface electromyography, maximal voluntary isometric contractions, the time to up and go, or the straight leg raise test. It is unclear how these parameters translate into clinical practice. In the context of outpatient surgery, readiness for discharge is an important general outcome, including various factors such as pain management, associated side effects, mobility, vital parameters, and the presence of any surgical complications such as bleeding [18]. We compared the impact of ACB versus FNB on readiness for discharge in patients undergoing outpatient ACL reconstruction. We hypothesized that ACB would lead to adequate pain control with preserved motor strength and a favorable safety profile, thereby facilitating earlier readiness for discharge.

## 2. Materials and Methods

### 2.1. Study Design

This multi-center, pragmatic, single-blinded, parallel-grouping superiority trial was conducted at a tertiary referral center (University Medical Center Amsterdam) and a secondary referral center (Dijklander hospital, Hoorn) in the Netherlands. The study was approved by the Dutch Medical Ethics Committee (#46184 on 20 January 2014) and adhered to the Declaration of Helsinki (Fortaleza, Brazil, 2013), as well as Good Clinical Practice guidelines and the General Data Protection Regulations. The study was registered before the inclusion of the first subject (https://clinicaltrials.gov/study/NCT02071433?cond=NTC020271433&rank=1, accessed on: 25 February 2014).

### 2.2. Patients

Adult patients, American Society of Anesthesiologists (ASA) classification I–III scheduled for elective outpatient ACL reconstruction were assessed for eligibility. Exclusion criteria were BMI > 35, an infection at the site of injection, coagulopathies, allergy to local anesthetics, pre-existing diagnosed neuropathy of the operated leg, chronic opioid use, pregnancy or breastfeeding status, any history of significant cardiovascular disease (myocardial infarction, cerebrovascular accident, or peripheral vascular disease), and inability to provide oral and written informed consent.

### 2.3. Randomization and Masking

Patients were randomly allocated in a 1:1 ratio to either the FNB or ACB group employing an online computer-based system with block randomization (size of four). As per the study’s nature, neither the patient nor the anesthesiologist performing the block were blinded. Nevertheless, the outcome observer, who conducted the sensory block testing, was blinded, and the data were collected on a separate case report form to maintain the research assistant’s blindness to the study group allocation. 

### 2.4. Procedures

All patients were administered premedication with paracetamol (2 g p.o.) and diclofenac (100 mg p.o.), unless contraindicated. An intravenous cannula was inserted in the forearm and infusion of Sterofundin^®^ ISO or Ringer-lactate (B. Braun, Oss, The Netherlands) was initiated at a rate of 3 mL/kg/hour. Both groups underwent ultrasound-guided nerve blockade prior to the induction of general anesthesia. Nerve block was performed by an experienced anesthesiologist. The spread of local anesthetic was recorded by employing ultrasound. All patients received general anesthesia, using a laryngeal mask airway and propofol target controlled infusion (effect site concentration between 2.5–3.5 μg/mL, approximately 6–9 mg/kg/h) and remifentanil infusion (15–23 mcg/kg/h). Subsequently, all patients underwent arthroscopic all-inside ACL reconstruction with a hamstring or quadriceps tendon autograft. A tourniquet was used in all patients. As for the consistency of performing the surgical procedure, two orthopedic surgeons, fellowship-trained in traumatology and sports-medicine, performed all procedures. During surgery, a morphine bolus of 0.1 mg/kg was administered, and all patients received dexamethasone in a dose of 4 mg intravenously. After surgery, patients were transferred to the post-anesthesia care unit (PACU). Morphine (2 mg i.v.) was titrated until patient-reported pain levels were ≤4 on the numeric rating scale (NRS). Postoperative pain management consisted of paracetamol (1000 mg orally every 6 h) and diclofenac (50 mg orally every 8 h) for both groups. Upon discharge, patients were provided with prescriptions for paracetamol (1000 mg orally every 6 h), diclofenac (50 mg orally every 8 h), and oxycodone (5 mg orally every 6 h), as required. 

Femoral nerve block (control): The femoral nerve was identified lateral to the femoral artery and superficial to the iliopsoas muscle as described before [19]. The block was initiated with 15 mL levobupivacaine 0.5% (Astra Zeneca BV, The Hague, The Netherlands), after needle placement using an in-plane technique. 

Adductor canal block (intervention): The transducer was transversely placed over the medial part of the mid-thigh and was slid distally to identify the femoral artery and vein subsartorially. The border of the sartorius (anterolateral) and adductor longus muscle (posterior) can be identified. From this point, the apex of the femoral triangle is proximally limited. The injection target is distal from the femoral triangle in the beginning of the adductor canal. Using a 50–80 mm gauge echogenic needle, 15 mL of levobupivacaine 0.5% (Astra Zeneca BV, The Hague, The Netherlands) was administered using an in-plane technique. 

### 2.5. Outcome

The primary outcome was the difference between groups in terms of readiness for discharge, as measured by the Post-Anesthetic Discharge Scoring System (PADSS). The PADSS is a valid and reliable scoring system that assesses various aspects of safe discharge, including vital signs, ambulation, nausea/vomiting, pain, bleeding and urinating (Online Supplementary Material A) [18]. Each item was graded from 0 (worst) to 2 (best) and then summed up. A score of ≥9 indicates that the patient is ready for discharge. 

Secondary outcomes included between-group differences in short-term outcome measures, i.e., the incidence of motor block, as measured by the Medical Research Council (MRC) scale, [20] the incidence of (near) falls, time to mobilization, assessment of sensory block one hour after surgery, the Overall Benefit of Analgesia Score (OBAS) on postoperative day (POD) 0 and 1, total postoperative opioid consumption at the PACU and on POD 1 at home, and NRS scores during hospitalization. Furthermore, long-term outcomes at 6 and 12 weeks were (near) falls, neurology of the knee assessed by the orthopedic surgeon, the Medical Outcomes General Health Survey (SF-36), and Knee Injury and Osteoarthritis Outcome Score (KOOS).

### 2.6. Measurements

The PADSS score was evaluated every 30 min after surgery. The incidence of motor block was measured one hour after surgery by the Medical Research Council (MRC) scale, which ranges from zero to five, with scores below four indicating motor block [20]. The frequency of falls, including both actual falls and near falls, that occurred during inpatient active mobilization was recorded prior to discharge. Furthermore, during a follow-up call on POD 1, the outcome assessor inquired specifically about the occurrence of any falls or near falls within the past 24 h. A near fall was defined as an occurrence of stumbling or experiencing a momentary loss of balance, which would lead to an actual fall if the necessary recovery mechanisms were not activated [21]. We defined a fall according to the definition of Lach et al.: “an unexpected loss of balance resulting in coming to rest on the floor, the ground, or an object below knee level [22].” Sensory block area was assessed one hour after surgery at both lateral and medial sides of the ankle and knee. Opioid consumption was measured in oral milligram morphine equivalents (MMEs) (online Supplementary Material A). The OBAS score is a validated patient-reported outcome measure of pain that, besides pain intensity, includes opioid side effects and patient satisfaction [23]. It ranges from 0 (best) to 28 (worst) and was measured on POD 0 and POD 1. The NRS score was used to assess pain every 30 min during hospitalization until a maximum of 10 h after surgery.

The SF-36 questionnaire was used to assess generic health status before surgery and at 6 and 12 weeks after surgery. It includes eight subscales: physical functioning, physical role limitation, emotional role limitation, energy/fatigue, emotional well-being, social functioning, pain, and general health [24]. Each subscale was individually calculated and transformed by subtracting the minimum score from the raw score, and dividing this by the subscale range, multiplied by 100. This calculation results in a score between 0 (extreme problems) and 100 (no problems) [25].

The KOOS questionnaire, another reliable and valid outcome measure that assesses patient relevant outcomes after knee injury, was taken before surgery, and 6 and 12 weeks after surgery. Five subscales were evaluated including pain, symptoms, activities of daily living, sport and recreation function, and knee-related quality of life [26]. Each subscale was individually calculated and transformed by dividing the raw score by the maximum score and multiplied by 100. This number was subtracted from 100, which resulted in a score between 0 (extreme knee problems) and 100 (no knee problems). 

### 2.7. Statistical Analysis

#### Sample Size

To detect a reduction in readiness for discharge of at least one hour (using 1.2 h in our calculation), estimating a standard deviation of 1.5 h with a power of 80% and a two-sided alpha of 0.05, we needed 26 patients per group.

Continuous data were described as medians and interquartile ranges or means with standard deviations depending on the distribution of the data, which was assessed by visual inspection. Categorical data were described with numbers and percentages. According to the intention-to-treat principle, among all randomized patients, Kaplan–Meier survival curves were used to analyze the time to readiness for discharge. A log-rank test was performed to test for differences between groups [27]. To assess differences between groups in the incidence of motor blocks, sensory blocks, or (near) falls, we used Fisher’s exact test. Differences in opioid consumption and OBAS score were analyzed using the Wilcoxon Rank test. To analyze NRS scores over time, we used a mixed-effects model. First, a baseline linear model was fitted with NRS as the dependent variable and time and randomization group as the fixed effect with a random intercept and slope. Thereafter, an interaction term (time*randomization) was added to the model. Pairwise deletion was used to analyze patient-reported outcome measures (PROMs) to maximize all data available. The SF-36 and KOOS scores were analyzed using a Student’s *t*-test. A two-sided *p*-value < 0.05 was considered statistically significant. All statistical analyses were performed using R studio (Affero General Public License V3).

## 3. Results

**Figure 1:**
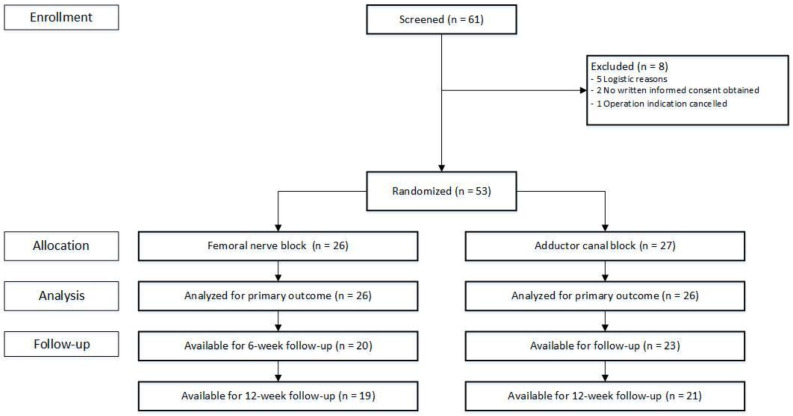
Flow diagram.

From March 2014 to July 2017, 61 patients were screened for participation in this trial. Fifty-three patients provided written informed consent. Of these, 26 patients were randomly allocated to receive FNB, and 27 to receive an ACB. The time to PADSS score indicating readiness for discharge was available for 52 (98.1%) patients, with 43 (81.1%) patients available at the 6-week follow-up and 40 (75.5%) completing the 12-week follow-up (Figure 1). 

A minority of patients were female (35.8%) and the median age of patients was 27 [IQR 21, 37]. The median NRS score before surgery was 0 [0, 0] in rest and 0.5 [0, 4] during movement. Patient characteristics are shown in groups (Table 1).

### 3.1. Primary Outcome

**Figure 2:**
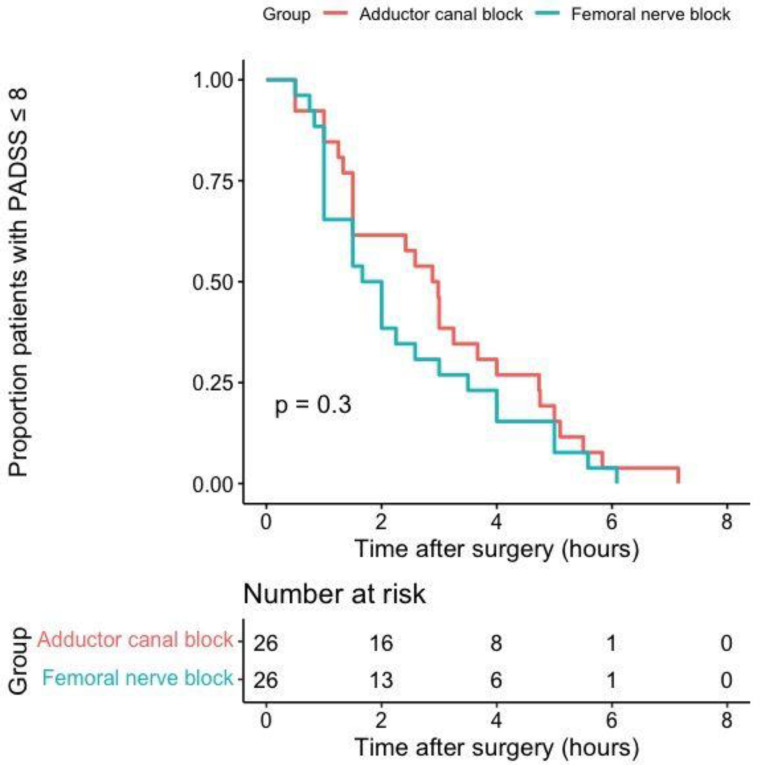
Between-group differences in readiness to home discharge estimated by Kaplan–Meier method. Kaplan–Meier curves displaying the estimated proportion of patients ready for discharge (i.e., PADSS ≥ 9) for patients allocated to the adduct or canal block or femoral nerve block. Each vertical step in the curve indicates a patient ready for discharge. The log-rank test indicates no difference between the survival curves. Abbreviations: PADSS = Post-Anesthetic Discharge Scoring System.

No between-group difference in readiness for discharge was found (FNB median 1.8 (95% CI 1.0 to 3.5) hours vs. ACB 2.9 (1.5 to 4.7) hours, *p* = 0.3 (Figure 2). 

### 3.2. Secondary Outcomes

In 20 (76.9%) patients with FNB vs. 1 (3.7%) patient with ACB, a motor block (MRC < 4) was recorded (*p* < 0.001, Table 2). A (near) fall was reported in 7 (29.2%) patients with FNB compared to 1 (4%) patient with ACB (*p* = 0.023). The majority of patients with FNB (84%), compared to all patients with ACB (100%), were able to mobilize within 24 h (*p* = 0.112). The time to first mobilization was similar between groups (*p* = 0.1). Distribution of sensory block was similar between groups (Online Supplementary Material B).

Patients with FNB consumed less opioids in the PACU compared to patients allocated to ACB (median 3 [0, 21] vs. 15 [12, 42.5] oral MME, *p* = 0.004). However, on postoperative day 1, opioid consumption was similar between groups (median 2.5 [2, 4.8] vs. 2 [1, 4] oral MME, *p* = 0.527. The OBAS score was comparable between groups on POD 0 and POD 1. No difference between groups was found in postoperative NRS scores over time (*p* = 0.124, Online Supplementary Material C). No differences were found at the 6- or and 12-week follow-up regarding (near) falls, nor neurology of the knee as assessed by the orthopedic surgeon. Long-term patient-reported outcome measurements did not differ between groups (Table 3). 

## 4. Discussion

In this RCT evaluating the effectiveness of ACB as compared to FNB in patients undergoing ACL reconstruction, we detected no significant difference in readiness for discharge between ACB and FNB. Our findings did suggest that FNB may provide superior pain relief on the day of surgery. Conversely, motor block was more prevalent in patients receiving FNB, which was accompanied by an increased risk of potential falls. No differences in long-term outcomes were observed.

Varying outcomes have been reported on readiness for discharge between ACB and FNB in patients undergoing ACL reconstruction. In agreement with our study, Seanglueulur et al. reported a comparable readiness for discharge between groups [14], whereas Abdallah et al. found a shorter time to discharge by 18 min in patients with ADC compared to FNB [8]. The authors attributed this finding to the earlier achievement of ambulation criteria, as assessed by the Aldrete score. Although Abdallah’s results showed a statistical difference, one may wonder if this difference was also clinically relevant. 

We observed a higher incidence of both motor blocks and potential falls in patients receiving FNB compared to ACB. The literature presents varying findings regarding the impact of nerve block on quadriceps strength. Several studies have reported reduced quadriceps strength, or a higher risk of falls in patients with FNB compared to ACB [8,9,10,11,12]. However, three RCTs involving 197 patients did not find any statistically significant differences in quadriceps strength, nor recovery of knee function [15,16,17]. Studies investigating functional recovery have shown substantial heterogeneity in the utilized outcome parameters and the timing of measurement. This heterogeneity complicates a direct comparison between studies. Nevertheless, it appears evident that ACD provides superior motor-sparing effects across various outcomes compared to FNB.

In our study, patients with FNB consumed less opioids during their PACU stay compared to patients with ACB. This contrasts from the findings of Sullivan et al., who reported a similar opioid consumption during PACU phase in 86 patients undergoing ACL reconstruction allocated to FNB or ACB [10]. A similar opioid administration during the PACU period aligns with the conclusion drawn in the Cochrane review conducted by Schnabel et al., which involved a meta-analysis of five trials comprising 305 patients undergoing knee surgery [28]. It is important to note that in our study, the decrease in opioid usage in the FNB-group did not persist beyond PACU stay. Furthermore, the increase in morphine consumption among ACB patients did not result in a deterioration in OBAS score on the day of surgery. Both of these findings necessitate a critical evaluation of the clinical relevance of this slight decrease in morphine consumption in patients with FNB within the context of our study.

We were surprised by the relatively frequent occurrence of sensory block in the lateral ankle. Since we assessed sensory perception immediately after surgery in the PACU, our hypothesis was that the tourniquet application may have induced altered sensory experiences in the patients.

This study has some limitations. Patients were recruited at a slower-than-anticipated rate over a period of three years, primarily due to logistical challenges. Furthermore, we encountered a relatively high rate of missing data (70–75% available) for PROMs at 6 and 12 weeks of follow-up. We did not impute the missing data. We acknowledge that this lack of imputation will decrease the statistical power; however, PROMs are part of our secondary outcome and should be interpreted as exploratory in nature. Another potential limitation is that both the treating physicians and patients were not blinded to group allocation, which could have introduced bias. However, we took measures to mitigate bias by ensuring that the outcome assessor remained blinded throughout the study. Finally, we acknowledge the time gap between the surgical procedures and the dissemination of the findings. It is important to realize that medical knowledge and surgical techniques could have changed during this period. Accordingly, when interpreting the findings within their contextual framework, this publication retains the capacity to furnish valuable insights.

The strengths of this study include the multi-center, randomized trial design. Instead of focusing on a specific traditional outcome such as pain score, we chose comprehensive outcomes such as readiness for discharge and PROMs. Additionally, we studied long-term outcomes, an aspect that tends to be insufficiently explored in these types of studies.

## 5. Conclusions

In this RCT, ACB did not shorten the time to readiness for discharge as compared to FNB in patients undergoing ACL reconstruction. Nevertheless, ACB may be considered the preferred technique due to a higher occurrence of motor block and potential falls in patients receiving FNB. Additionally, the slight increase in morphine consumption in patients receiving ACB immediately after surgery did not lead to a worsened overall pain control, taking into account the OBAS, a PROM that incorporates satisfaction and side effects alongside the traditionally assessed pain intensity.

## Figures and Tables

**Table 1:** Patient baseline characteristics.

	FNB (*n* = 26)	ACB (*n* = 27)
Female sex	9 (34.6%)	10 (37.0%)
Age, years ^a^	27.0 [21.3, 40]	25.0 [21, 36]
BMI, kg·m^−1 a^	25.2 [22.5, 27.0]	23.8 [21.7, 26.7]
Smoking	5 (19.2%)	13 (48.1%)
Pain intensity before surgery		
NRS score at rest ^b^	0 [0, 0]	0 [0, 0]
NRS score on movement ^b^	1 [0, 4]	0 [0, 3]
Surgical details		
Length of surgery, in minutes ^a^	56.5 [47.5, 65.0]	59.0 [53.5, 72.5]
Choice of graft		
Hamstring tendon autograft	24 (92.3%)	23 (85.2%)
Quadriceps tendon autograft	0	2 (7.4%)
Unknown	2 (7.7%)	2 (7.4%)

Variable distributions were reported as number and percentage unless specified otherwise. a = Median [IQR]. b = Mean (SD). Abbreviations: FNB = femoral nerve block, ACB = adductor canal block, BMI = body mass index, NRS = numeric rating scale.

**Table 2:** Secondary outcomes.

	Available for	FNB (*n* = 26)	ACB (*n* = 27)	*p*-Value
Incidence of a motor block, 1 h after surgery ^a^	48 (90.6%)	20 (76.9%)	1 (3.7%)	<0.001 ***
The incidence of (near) falls first 24 h	49 (92.5%)	7 (29.2%)	1 (4%)	0.023 *
Mobilization				
Able to walk first 24 h	49 (92.5%)	22 (84.6%)	23 (100%)	0.112
Time to first mobilization, hours ^b^	36 (67.9%)	19.0 (7.8, 23.1)	19.0 (17.4, 26.66)	0.100
Opioid consumption				
Total on PACU, in oral MME ^c^	53 (100%)	3.0 [0, 21.8]	15.0 [12.0, 42.5]	0.004 **
Total POD 1, in oral MME ^c,d^	51 (96.2%)	0 [0, 7.5]	0 [0, 7.5]	0.527
OBAS ^c^				
Day of surgery	52 (98.1%)	3.0 [1.0, 6.5]	4.0 [2.0, 5.8]	0.402
Postoperative day 1	53 (100%)	2.5 [2.0, 4.8]	2.0 [1.0, 4.0]	0.220
Week 6				
(Near) falls	40 (75.5%)	2 (10.5%)	-	
Global intact neurology knee ^e^	43 (81.1%)	11 (55%)	11 (47.8%)	0.763
Sensations of neuropraxia ^e^	40 (75.5%)	10 (50%)	13 (65%)	0.197
Week 12				
(Near) falls	38 (71.7%)	1 (5.3%)	-	
Global intact neurology knee ^e^	40 (75.5%)	10 (52.6%)	6 (28.6%)	0.523
Sensations of neuropraxia ^e^	39 (74.6%)	8 (42.1%)	11 (55%)	0.527

Variable distributions were reported as number and percentage unless specified otherwise; Fisher’s exact test. * *p* ≤ 0.05, ** *p* ≤ 0.01, *** *p* ≤ 0.001. a: The incidence of a motor block as defined as a Medical Research Council scale score < 5. b: Median (95% confidence limit); log-rank test. c: Median [IQR]; Wilcoxon Rank Sum. d: Measured until 16:00 on POD 1. e: Assessed by the orthopedic surgeon. Abbreviations: FNB = femoral nerve block; ACB = adductor canal block; PACU = post-anesthesia care unit; MME = milligram morphine equivalent; OBAS = Overall Benefit of Analgesic Score.

**Table 3:** Patient-reported outcome measurements.

	Available for	FNB (*n* = 26)	ACB (*n* = 27)	*p*-Value
Baseline				
SF-36				
Physical functioning		73.4 (17.1)	76.6 (20.6)	
Role limitations due to physical health		44 (39.7)	68 (37.2)	
Role limitations due to emotional problems		78.7 (34.5)	86.7 (27.2)	
Energy, fatigue		70.4 (16.1)	78 (13.8)	
Emotional well-being		77.6 (18.3)	83.7 (13.5)	
Social functioning		72.5 (23.1)	85.0 (18.8)	
Pain		66.4 (20.5)	76.1 (22.8)	
General Health		81.5 (17.0)	84 (15.3)	
KOOS				
Symptoms		64.0 (19.7)	75.4 (17.0)	
Pain		72.2 (20.7)	86.1 (20.9)	
Function in daily life		79.6 (18.0)	87.8 (13.4)	
Function in spare time and sport activities		41.5 (29.1)	45 (28.7)	
Knee-associated quality of life		39.8 (21.3)	53.9 (23.4)	
Week 6				
SF-36				
Physical functioning	41 (77.4%)	54.5 (22.5)	66.8 (14.4)	0.049 *
Role limitations due to physical health	40 (75.5%)	12.5 (15.5)	19.3 (31.7)	0.382
Role limitations due to emotional problems	41 (77.4%)	84.2 (34.0)	72.7 (42.0)	0.339
Energy, fatigue	41 (77.4%)	70.8 (14.9)	67.3 (16.2)	0.475
Emotional well-being	39 (73.6%)	84.9 (10.8)	77.3 (14.7)	0.072
Social functioning	41 (77.4%)	69.7 (22.6)	65.9 (21.2)	0.581
Pain	39 (73.6%)	58.9 (30.5)	67.4 (25.5)	0.358
General Health	41 (77.4%)	79.2 (13.9)	80.5 (14.2)	0.779
KOOS				
Symptoms	41 (77.4%)	59.6 (20.3)	69.2 (14.5)	0.092
Pain	41 (77.4%)	74.3 (22.0)	78.2 (18)	0.542
Function in daily life	38 (71.7%)	77.9 (19.9)	73.9 (22.9)	0.568
Function in spare time and sport activities	34 (64.2%)	33.1 (30.9)	34.4 (17.1)	0.881
Knee-associated quality of life	35 (66.0%)	47.9 (20.0)	59.9 (26.9)	0.146
Week 12				
SF-36				
Physical functioning	36 (67.9%)	77.6 (18.7)	78.2 (17.9)	0.921
Role limitations due to physical health	37 (69.8%)	63.2 (45.2)	48.6 (41.5)	0.315
Role limitations due to emotional problems	37 (69.8%)	94.7 (22.9)	87.0 (32.6)	0.415
Energy, fatigue	35 (66.0%)	76.7 (16.2)	68.8 (14.0)	0.134
Emotional well-being	34 (64.2%)	86.6 (7.7)	83.8 (15.5)	0.509
Social functioning	36 (67.9%)	88.8 (16.1)	87.5 (16.5)	0.811
Pain	37 (69.8%)	78.6 (20.5)	77.9 (15.2)	0.915
General Health	34 (64.2%)	78.6 (12.9)	79.1 (16.0)	0.929
KOOS				
Symptoms	36 (67.9%)	75.2 (17.5)	73.8 (11.9)	0.783
Pain	39 (74.6%)	85.0 (19.7)	87.0 (10.6)	0.695
Function in daily life	38 (71.7%)	87.2 (20.0)	86.1 (13.4)	0.835
Function in spare time and sport activities	37 (69.8%)	58.7 (27.8)	55.8 (22.3)	0.732
Knee-associated quality of life	38 (71.7%)	62.5 (19.3)	64.2 (22.2)	0.800

Variable distributions were reported as mean (SD); student’s *t*-test. * *p* ≤ 0.05. Abbreviations: FNB = femoral nerve block; ACB = adductor canal block; SF = Short Form Health Survey; KOOS = Knee Injury and Osteoarthritis Outcome Score.

## Data Availability

The data that support the findings of this study are available from the corresponding author upon reasonable request.

## Supplementary Materials

The following supporting information can be downloaded at: https://www.mdpi.com/article/10.3390/jcm12186019/s1, Online Supplementary Material A: Post-Anesthetic Discharge Scoring System [18]. Total PADS score is 10; Score ≥ 9 considered fit for home discharge; ** PO = oral administration. Online Supplementary Material B: Assessment sensory block. Variable distributions were reported as number and percentage unless specified otherwise. Abbreviations: FNB = femoral nerve block, ACB = adductor canal block. Online Supplementary Material C: Plot individual Numeric Rating Scale scores after surgery. No difference between groups was found in postoperative NRS scores over time (*p* = 0.124).
