# Supplementary material for: The Effectiveness of Adductor Canal Block Compared to Femoral Nerve Block on Readiness for Discharge in Patients Undergoing Outpatient Anterior Cruciate Ligament Reconstruction: A Multi-Center Randomized Clinical Trial
Source: J Clin Med. 2023 Sep 17;12(18):6019. doi: 10.3390/jcm12186019 (PMC10531554; doi:10.3390/jcm12186019)
Supplement: Supplementary file 1 [file jcm-12-06019-s001.zip › Online Supplementary Material A.pdf]

## Online supplementary material A: Post Anesthetic Discharge Scoring System[18]

| | |
|---|---|
| **Vital Signs** | 2 = within 20% of preoperative value<br>1 = 20%-40% of preoperative value<br>0 = > 40% preoperative value |
| **Activity and mental status** | 2 = Oriented × 3 AND has a steady gait<br>1 = Oriented × 3 OR has a steady gait<br>0 = Neither |
| **Pain, nausea and/or vomiting** | 2 = Minimal<br>1 = Moderate, having required treatment<br>0 = Severe, requiring treatment |
| **Surgical bleeding** | 2 = Minimal<br>1 = Moderate<br>0 = Severe |
| **Intake and output** | 2 = has had PO fluids AND voided<br>1 = has had PO fluids OR voided<br>0 = Neither |

*Total PADS score is 10; Score ≥ 9 considered fit for home discharge; **PO = oral administration.
