# Supplementary material for: The Effectiveness of Adductor Canal Block Compared to Femoral Nerve Block on Readiness for Discharge in Patients Undergoing Outpatient Anterior Cruciate Ligament Reconstruction: A Multi-Center Randomized Clinical Trial
Source: J Clin Med. 2023 Sep 17;12(18):6019. doi: 10.3390/jcm12186019 (PMC10531554; doi:10.3390/jcm12186019)
Supplement: Supplementary file 1 [file jcm-12-06019-s001.zip › Online Supplementary Material B_Revised.pdf]

Online supplementary material B: Assessment sensory block

	FNB (n = 26)	ACB (n = 27)
Medial knee		
No sensory block	6 (23.1%)	8 (29.6%)
Sensory block	19 (73.1%)	18 (66.7%)
Unknown	1 (3.8%)	1 (3.7%)
Lateral knee		
No sensory block	16 (61.5%)	12 (44.4%)
Sensory block	9 (34.6%)	14 (51.9%)
Unknown	1 (3.8%)	1 (3.7%)
Medial ankle		
No sensory block	8 (30.8%)	7 (25.9%)
Sensory block	17 (65.4%)	20 (74.1%)
Unknown	1 (3.8%)	-
Lateral ankle		
No sensory block	10 (38.5%)	16 (59.3%)
Sensory block	6 (23.1%)	6 (22.2%)
Unknown	10 (38.5%)	5 (18.5%)

Variable distributions were reported as number and percentage unless specified otherwise.
Abbreviations: FNB = femoral nerve block, ACB = adductor canal block.
