# Supplementary figures and images for: The Effectiveness of Adductor Canal Block Compared to Femoral Nerve Block on Readiness for Discharge in Patients Undergoing Outpatient Anterior Cruciate Ligament Reconstruction: A Multi-Center Randomized Clinical Trial
Source: J Clin Med. 2023 Sep 17;12(18):6019. doi: 10.3390/jcm12186019 (PMC10531554; doi:10.3390/jcm12186019)

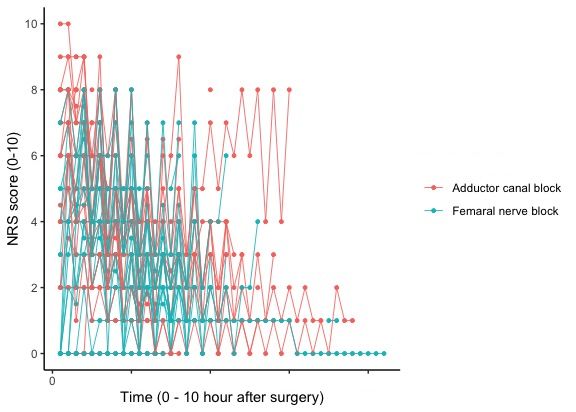

Supplement: Supplementary file 1 [file jcm-12-06019-s001.zip › Online Supplementary Material C_Revised.jpg]
